# The Role of Botulinum Neurotoxin A in the Conservative Treatment of Fractures: An Experimental Study on Rats

**DOI:** 10.1155/2024/7446251

**Published:** 2024-05-31

**Authors:** Themistoklis Vampertzis, Christina Barmpagianni, Chryssa Bekiari, Georgia D. Brellou, Ioannis A. Zervos, Eleftherios Tsiridis, Nikiforos Galanis

**Affiliations:** ^1^Faculty of Medicine, School of Health Sciences, Aristotle University of Thessaloniki, Thessaloniki, Greece; ^2^Surrey and Borders Partnership NHS Foundation Trust, Leatherhead, UK; ^3^Laboratory of Anatomy and Histology, Faculty of Veterinary Medicine, School of Health Sciences, Aristotle University of Thessaloniki, Thessaloniki, Greece; ^4^Department of Pathology, Faculty of Veterinary Medicine, School of Health Sciences, Aristotle University of Thessaloniki, Thessaloniki, Greece; ^5^Faculty of Veterinary Medicine, School of Health Sciences, Aristotle University of Thessaloniki, Thessaloniki, Greece; ^6^Academic Orthopaedic Department, Papageorgiou General Hospital and CORE Laboratory at CIRI-AUTH, AUTH Medical School, Thessaloniki, Greece

## Abstract

This paper explores the role of botulinum neurotoxin in aiding fracture recovery through temporary muscle paralysis. Specifically, it investigates the effects of botulinum neurotoxin-induced paralysis of the sternocleidomastoid muscle on clavicle fractures in rats. The research aims to assess safety, effectiveness, and the impact on fracture healing. Healthy male Albino Wistar rats were divided into four groups: clavicle fracture, botulinum neurotoxin injection, both, and control. Surgeries were conducted under anaesthesia, and postoperatively, animals were monitored for 28 days. Euthanasia and radiological assessment followed, examining fracture healing and muscle changes, while tissues were histopathologically evaluated. The modified Lane–Sandhu scoring system was used for the radiographic evaluation of clavicle fractures, and the results varied from complete healing to nonunion. Histopathological examination at 28 days postfracture showed fibrous tissue, mesenchymal cells, and primary callus formation in all groups. Despite varied callus compositions, botulinum neurotoxin administration did not affect clavicle healing, as evidenced by similar scores to the control group. Several studies have explored botulinum neurotoxin applications in fracture recovery. Research suggests its potential to enhance functional recovery in certain types of fractures. Theoretical benefits include managing muscle spasticity, aiding reduction techniques, and preventing nonunion. However, botulinum neurotoxin's transient effect and nonuniversal applications should be considered. The present study found that botulinum toxin had no clear superiority in healing compared to controls, while histological evaluation showed potential adverse effects on muscle tissue. Further research is essential to understand its risk-benefit balance and long-term effects.

## 1. Introduction

The management of fractures and the subsequent recovery process are evolving areas in the field of orthopedics. A bone fracture is defined as any break in bone continuity, and it is followed by a sequence of countermeasures to achieve healing through the formation of a bony callus [1, 2]. The stability of fracture parts is of major importance for callus formation, with unstable fractures posing the risk of nonunion or delayed union and the formation of pseudoarthrosis [1].

The traditional approach to fracture treatment focuses primarily on reduction via closed manipulation or open (surgical) fixation, followed by immobilization and physical therapy [2, 3]. A primary principle regardless of the open or closed approach is the maintenance of stability [3]. A key role in this is played by the action of the muscles around the fracture, which can displace it [4]. In conservative treatment, the need to reduce muscle action on the fracture site is greater as there are no osteosynthesis materials to maintain the reduced position [4]. This is primarily achieved by immobilization and requires the patients' compliance and efforts to limit movement [2–4]. However, research has experimented with innovative strategies to enhance healing, and recent advancements have introduced the use of botulinum neurotoxin [5, 6].

Botulinum neurotoxin (BoNT), commonly known as Botox, is a potent neurotoxic protein produced by the bacterium of the genus Clostridium [7]. Its main action is to prevent the release of the neurotransmitter acetylcholine from the nerve terminals at the neuromuscular junction, by selectively binding to the presynaptic membrane of motor neurons [7]. This inhibition interrupts the transmission of nerve impulses to the muscle, resulting in paralysis [6, 7]. This paralysis is temporary, and its duration depends on factors such as the type of toxin, dose, recipient organism, route of administration, and the type of nerve terminal [7]. Moreover, the effect is localized; thus, muscle relaxation/paralysis can be targeted [7].

Adverse effects associated with the use of BoNTs are normally limited to the injection site and include pain and irritation [7, 8]. Nevertheless, serious complications such as iatrogenic botulism have also been reported [7]. There are seven categories of botulinum neurotoxins known today, A-G, classified according to their characteristics [7]. BoNT A1 is considered the most effective as well as the safest one, which is why it is the most utilized in medicine [7].

Due to the above qualities, Botox has gained immense popularity for its cosmetic applications; however, its therapeutic potential extends beyond aesthetics and has found utility in various medical fields, including orthopedics. Experimental studies on rats have investigated the effect of botulinum toxin injected into the quadriceps muscle on the healing of femur fractures [8]. In addition, the action of botulinum toxin has been studied in cases of ectopic ossification following fractures, showing promising results [9].

This paper explores the applications of botulinum neurotoxins in temporarily paralyzing muscles to aid in fracture recovery and healing. Specifically, it examines the effects of botulinum neurotoxin A-induced paralysis of the sternocleidomastoid (STM) muscle on the healing of clavicle fractures in Wistar rats.

Clavicle fractures account for 2.6–10% of all adult fractures in the general population, while they are the most common fractures in the paediatric population [10, 11]. They are frequent in athletes, with having the third longest recovery time and return to the field posttraumatically, with 20% never returning [11]. In the general population, conservative treatment seems to be preferable to surgery, while only 55% of athletes receive conservative treatment [10]. Fractures mostly occur in the middle third of the clavicle, which is the thinnest and least protected part of the bone due to the absence of ligaments [10, 11]. The muscle acting on this centric part of the clavicle is the STM, which constitutes a destabilizer by displacing the bone upwards and backwards [11]. The STM plays a leading role in head and neck movements [12, 13]. In humans, it arises from the handle of the sternum and the middle third of the clavicle with the two heads forming a common muscle in the mastoid process and upper cervical line [12]. The STM by its action contributes to posture, turning, tilting, and extension of the head, while its action on the sternum and clavicle provides support to the temporomandibular joint during mastication [12, 13]. It is innervated by the cervical plexus and the accessory nerve (11^th^ cranial nerve), and its dysfunction can result in different degrees of occipital craniocervical pain, as well as cervical dystonia [12–14]. The injection of BoNT into the STM for the treatment of torticollis is steadily gaining ground with many authors recommending it [15–17]. Similarly, the applications of toxins outside the area of aesthetics include the treatment of overactive bladder [18–20].

This study is the second part of the experimental series and follows the neurophysiological/electrophysiological study conducted on Wistar rats to determine the safe dose of toxin that completely paralyzes the STM muscle for a minimum of 28 days. This first experiment established that 4 international units (IU) were nontoxic (following the rule of 2 IU/100 g of body weight) and sufficient for the desired paralysis [21, 22]. In the second arm of the experimental protocol, 4 IU of the toxin was injected intramuscularly following a surgically induced clavicle fracture under direct vision. The effects of the toxin were tested against a control group injected with normal saline and a group that did not receive any additional treatment following the fracture. Additionally, a group receiving 4 IU of toxin only was included, to evaluate the effects on the muscle tissue independently of the presence of a fracture. Fracture healing and callus formation were examined by radiographic and later histological review. The aim was to determine whether the paralysis of the muscle acting on the fracture site had any effects on the healing process. A secondary aim of the experiment was to review the effects of the toxin on the muscle tissues at a histological level and determine whether any adversities occurred as a result of the toxin administration. This research can provide insight into the expanding application of BoNT in clinical practice, outside the scope of aesthetics, and review the safety and effectiveness of its use in orthopedics.

## 2. Materials and Methods

### 2.1. Animals and Treatments

This is a Level I experimental study, that was conducted on healthy adult male rats of the Albino Wistar breed, between 4 and 6 months of age, weighing between 350 and 450 grams. Rats were chosen because of the similarities of their anatomical configuration to that of humans [23]. Studies on the possible effect of gender on the effectiveness of botulinum toxin A muscle injection in rats are lacking; however, studies in humans revealed no difference in the dose and effectiveness of botulinum toxin A muscle injection between males and females [24]. To our knowledge, the majority of studies that examine muscle integrity and function after botulinum toxin A injection in rodents use male rats, which is the reason only male rats were included in the study [24].

The animals were divided into four groups of seven animals each (a total of 28 animals) and were marked accordingly for recognizing purposes. In Group A, a clavicle fracture was intraoperatively induced. In Group B, 4 IU BoNT was injected intramuscularly into the STM muscle. Group C underwent an intraoperative clavicle fracture and intramuscular injection of 4 IU BoNT into the corresponding STM muscle. Group D received an intramuscular injection of 0.9% normal saline (NaCl) in addition to the intraoperatively induced clavicle fracture. The groups are given in Table 1.

For the duration of the experiment, the animals were bred (accreditation number EL-54BIObre- 43) and kept at the animal facility of the Experimental and Research Centre of Papageorgiou General Hospital Thessaloniki (accreditation number EL-54-BIOexp-01) and consumed food and water ad libitum. Animals were group-housed under fully controlled conditions, and a light/dark cycle of 12 hours was followed.

The procedures were in full accordance with the European Community Council directive 86/609/EEC. Granting of a research protocol license by the Region of Central Macedonia, General Directorate of Agricultural Economy and Veterinary Medicine, Directorate of Veterinary Medicine, Department of Animal Health and Veterinary Perception, Medicines and Applications after applying for protocol authorization to the Directorate of Veterinary Medicine, Region of Central Macedonia and to the Protocol Evaluation Committee of the General Hospital Papageorgiou Research Center preceded the experiments. The research protocol experiment permit had the protocol number 497690/12/09/2019(2085). Finally, the instructions of the committee for the use and care of laboratory animals of the Aristotle University of Thessaloniki were followed.

In rats, the STM is composed of two bellies, a superficial sternomastoid medially and a deeper cleidomastoid laterally [25]. The surgeries were performed under general anaesthesia in aseptic conditions, by one surgeon. A mixture of ketamine/xylazine, 50, and 5 mg/kg was administered to induce general anaesthesia and was followed by cleaning and shaving the skin above the right STM muscle and the right clavicle. An approximately 1.5 cm incision was then made on the anterior surface of the clavicle.

Dissection of the STM muscle and the middle third of the clavicle were performed, protecting the surrounding soft tissues (nerves, vessels). A fracture was induced in the middle third of the clavicle with a straight sharp surgical scissor. The diameter of the clavicle in the middle third is ≈1.5 mm, which easily allows the fracture to be performed in this way. Animals in groups B and C received 4 IU botulinum toxin and were injected intramuscularly into the corresponding sternocleidomastoid muscle (with BD Micro-Fine + insulin syringes with an integrated, nondetachable needle). Botox type A toxin (BOTOX PD.INJ SOL 100U/VIAL BT X 1VIAL) was used, purchased from Allergan Pharmaceuticals Ltd, a substance approved by the National Medicines Agency (approved 24802.01.01). The skin was sutured with Nylon 3/0 ETHILON simple interrupted suture.

Postoperatively, all animals were examined daily by a trained veterinary professional for pain, shortness of breath, or discomfort. No abnormal findings were observed during this period, including unaltered feeding habits and behavior. However, paracetamol (DEPON, BristolMyersSquibb SA), at a dose of 1800 mg/kg/24 h, was administered for 3 days via their drinking water for the elimination of any nondetected postoperative pain. During the postoperative period, the animals were allowed to freely move in their cages and no immobilization of the treated forelimb occurred. This was due to the animals' need for the limb for feeding, which would have made obstructing its use unsafe.

On the 28th postoperative day, the animals were euthanized by carbon dioxide (CO_2_) inhalation, a method that causes minimal psychological stress and does not induce pain [26, 27]. A radiological review followed euthanasia at the radiological laboratory of the Faculty of Veterinary Medicine of the University of Thessaloniki to study the progress of fracture healing.

After the completion of the radiological control, the clavicles of the animals were dissected at the Experimental and Research Center of General Hospital Papageorgiou in Thessaloniki. They were sent to the Laboratory of Pathology, School of Veterinary Medicine, Faculty of Health Sciences, Aristotle University of Thessaloniki, for histopathological assessment of clavicle fracture healing and of potential changes in the adjacent STM muscle fiber bundles, due to injection with BoNT.

### 2.2. Tissue Preparation for Histopathological Assessment

Clavicles and STM muscle samples from all animals were fixed in 10% buffered formalin. Sequentially, clavicles were decalcified in hydrochloric acid and formalin-based solution (OSTEOFAST 1, Biognost, Zagreb, Croatia), washed in tap water and immersed in formalin. All tissue segments were routinely processed, embedded in paraffin wax, and 4–6 *μ*m thick sections were stained with hematoxylin and eosin. Blind histopathological evaluation was conducted by a single pathologist. Clavicular fracture healing/repair included scoring according to Huo et al., and the grading scale ranged from 1 to 10 [28].

STM muscle sections were also examined [28].

### 2.3. Statistical Analysis

All data were analyzed using SPSS 28.0 statistical software. One-way analysis of variance (ANOVA), followed by Tukey's post-hoc analysis, and independent samples *T*-test were used for comparisons between the groups. Homogeneity of variances was tested using Levene's test, and when violated, the nonparametric two-tailed Kruskal–Wallis test was used for multiple comparisons, followed by the Mann–Whitney *U* test for two-by-two comparisons. All results are expressed as the mean ± S.E.M. Significance was set at *P* < 0.05.

## 3. Results

### 3.1. Radiographic Assessment

**Figure 1:**
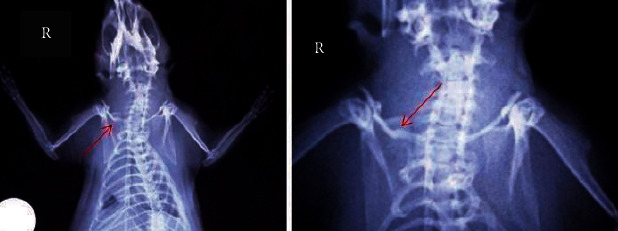
X-ray showing the right clavicle of an animal in group A (clavicle fracture only). The red arrow indicates callus formation, seen in magnification on the right. Modified Lane–Sandhu grade 7 considering bone formation occupying 75% (3 points), radiographic union (2 points), and proximal union (2 points).

**Figure 2:**
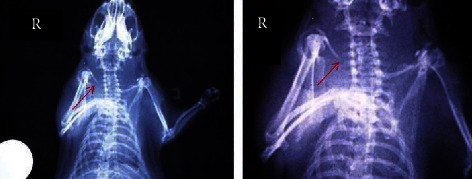
X-ray showing the right clavicle of an animal in group A (clavicle fracture only). The red arrow indicates the fracture site, seen in magnification on the right. Scoring 0 according to the modified Lane–Sandhu grading scale.

**Figure 3:**
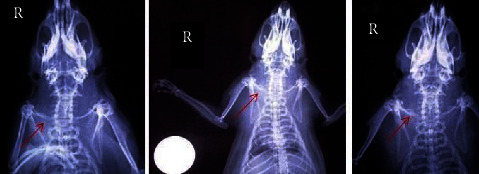
X-ray showing the right clavicle of an animal in group C (clavicle fracture plus 4 IU toxin). The red arrow indicates callus formation, seen in magnification on the right. Modified Lane–Sandhu grade 10.

**Figure 4:**
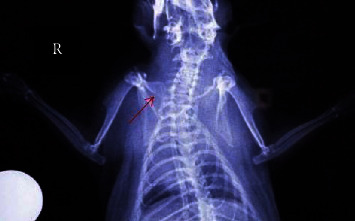
X-ray showing the right clavicle of the animal in group C (clavicle fracture plus 4 IU toxin). The red arrow indicates callus formation of grade 6 according to the modified Lane–Sandhu scale.

**Figure 5:**
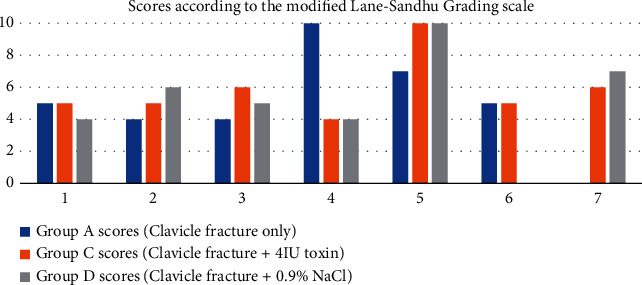
Scores based on the modified Lane–Sandhu grading scale following the radiological review of animals in groups A, C, and D.

The modified Lane–Sandhu scoring system was used for the radiographic assessment of clavicle fracture healing in animals treated with the toxin, in those injected with saline and in the group that did not receive any additional intervention (Figures 1–4) [29, 36]. Animal scores on radiological evaluation ranged from 0 (nonunion) to 10 (complete healing) and are described in detail in Figure 5.

### 3.2. Histopathological Evaluation of Clavicular Fracture Healing and of the Sternocleidomastoid Muscle

**Figure 6:**
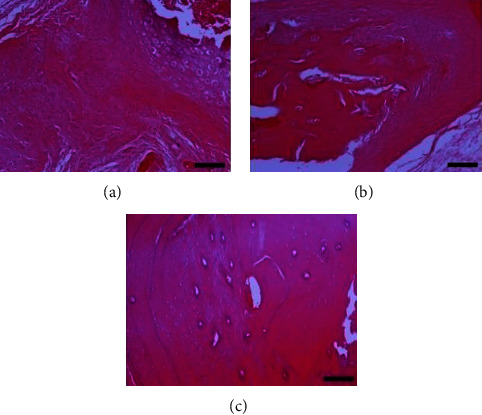
Clavicle tissue sections from animal subjects in group C (clavicle fracture plus 4 IU toxin). Images on the (a–c) show different degrees of healing according to the Huo scoring system. Hematoxylin and eosin, original magnification ×10. (a) Huo grade 5, bone cavity with a fragment of necrotic bone surrounded by a dense aggregate of chondrocytes. At the periphery of the aggregate, cartilaginous segments with either basophilic or eosinophilic matrix are observed. Woven bone with collagen varying in density as well as the presence of preosteoblasts and osteoblasts is also seen. Fibrous connective tissue was observed on the upper left end of the section. (b) Huo grade 8, thickened periosteum, and increased formation of woven bone rich in osteoblasts, expanding towards the mature bone. At the borders between mature and woven bone, sparse basophilic shift lines are prominent. (c) Huo grade 10, mature/lamellar compact bone with osteons, consisting of concentrically oriented lamellae and a central canal containing blood vessels. Osteocytes embedded in lacunae are also evident. Basophilic material (calcification) with random linear distribution located at the periphery of canals is observed.

**Figure 7:**
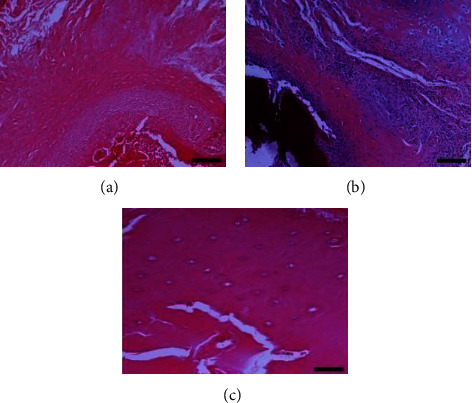
Clavicle tissue sections from animal subjects in group A (clavicle fracture only). Images on the (a–c) show different degrees of healing according to the Huo scoring system. Hematoxylin and eosin, original magnification ×10. (a) Huo grade 7, from bottom to top, are seen medullary cavities surrounded by immature chondrocytes/chondroblasts lying in an eosinophilic matrix, a crown consisting of a dense layer of mesenchymal stem cells/progenitor cells, and an extensive area of woven bone formation characterised by numerous osteoblasts and a dense, eosinophilic matrix with haphazardly arranged fibres. (b) Huo grade 7, extensive area on the upper right part of the section, containing chondrocytes lying in an eosinophilic and less frequently lightly basophilic matrix. Progenitor cells, mesenchymal cells, and pre- and/or osteoblasts are observed either dispersed or arranged in dense aggregates and are surrounded by an acidophilic matrix of varying degrees (immature/woven bone). The presence of connective tissue and vessels of small diameter are evident on the middle right end. In the lower left area, mature bone in necrotic phase is prominent. (c) Huo grade 10, mature/lamellar compact bone with osteons, consisting of concentrically oriented lamellae and a central canal containing blood vessels. Osteocytes embedded in lacunae are also obvious. Similarities are noted between this section and the one on Figure 6(c).

**Figure 8:**
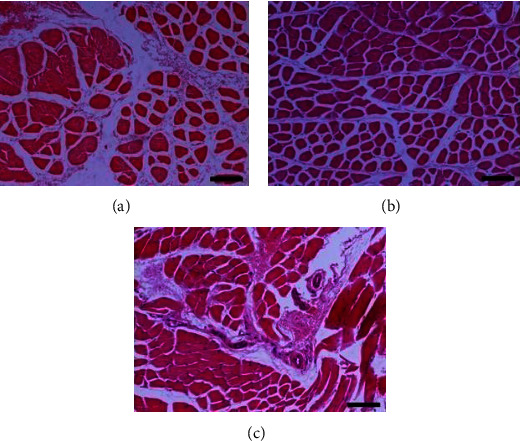
Muscle tissue sections from animal subjects in group B (4 IU toxin only). Images on the (a–c) show different degrees of healing according to the Huo scoring system. Hematoxylin and eosin, original magnification ×10. (a) Oedema of inter- and endomysium, myofiber degeneration, and atrophy characterised by muscle fibers showing rarefaction of myofibrils, size variation, and/or angular shape. Certain fibers are enlarged due to hypertrophy. (b) Oedema of inter- and endomysium appears less intense than in Figure 9. Additionally to atrophy, hypertrophic muscle fibers sometimes with longitudinal subdivision/splitting under the same endomysium are observed. (c) Hemorrhage of the perimysium and presence of myofibers with segmental necrosis (middle right). Necrotic fibers show intense eosinophilic sarcoplasm and absence of striation and pycnotic nuclei.

**Figure 9:**
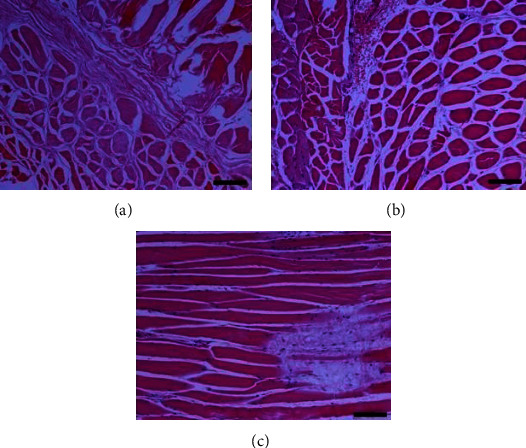
Muscle tissue sections from animal subjects in group D (0.9% NaCl only). Images on the (a–c) show different degrees of healing according to the Huo scoring system. Hematoxylin and eosin, original magnification ×10. (a) Endomysial oedema and multiple fibers showing myofibrillar rarefaction are noticed. Several myofibers present with intense eosinophilic and homogenous sarcoplasm, small size, and angular shape (atrophy). Perimysium and to a lesser extent endomysium are considerably prominent due to probable fibrosis and the absence and/or shrinkage of fibers. (b) Endomysium shows oedema and several fibers with atrophy and hypertrophy. Characteristic is the presence of a group of muscle fibers, with angular to irregular shape, intensely eosinophilic sarcoplasm, usually small in size and nuclei with pyknosis or karyorrhexis (necrosis) and hypercontraction of certain myofibers. The latter group presents a hemorrhage at its periphery. (c) A considerable number of fibers present mild depletion of myofibrils and loss of transverse striation. Note an extensive area where segments of adjacent muscle fibers are highly vacuolated, but the sarcolemma appears almost intact. In these regions, sarcoplasm is totally absent (lower right).

Histopathological evaluation of the fractured clavicles, at 28 days postfracture, revealed the presence of fibrous tissue, mesenchymal cells, osteoblasts, and chondroblasts in varying proportions (Figures 6(a) and 7(a)). The presence of woven bone mixed with cartilage (primary callus) was also observed. The primary callus consisted mainly of collagen fibres of irregular arrangement and was dominated by preosteoblasts and numerous large osteoblasts (Figures 6(b) and 7(b)). Only a small number of osteocytes were found, and calcium deposition was either absent or occurred to a small extent. The formation of the mature lamellar bone (secondary callus) was seen in only a few of the examined clavicles and consisted mainly of compact and less frequent trabecular bone (Figures 6(c) and 7(c)). In all experimental animals, regardless of botulinum toxin administration, bridging of the two bone ends was not complete. In addition, histopathological evaluation of the muscle tissue from groups with or without BoNT revealed marked differences, namely, necrosis and atrophy in the former group (Figures 8 and 9).

Following the scoring system of Huo et al. [28], the fractured clavicular area had a mean score of 7.18 ± 0.296 for the control animals and a mean score of 7.17 ± 0.386 for the animals that received a botulinum toxin injection, revealing that botulinum toxin administration on the STM muscle did not affect the healing process of fractured clavicles.

## 4. Discussion

Several studies have explored the use of botulinum neurotoxin in fracture recovery and healing. A study by Tukel et al. tested the effect of botulinum toxin type A on mandibular fracture healing in rabbits and demonstrated that BoNT injection into the masseter muscle improved bone healing [5].

Another study investigated the effects of botulinum neurotoxin in ankle fractures associated with Achilles tendon tightness [30]. The results indicated that BoNT injections reduced tendon tension, improved fracture alignment, and facilitated early weight-bearing, thereby accelerating the recovery process [30].

Research and literature aim to investigate other uses of Botox by extending its applications in fracture recovery, facilitation of reduction techniques, and prevention of nonunion [6, 9, 30]. The theoretical, potential benefit would be that in cases where fractures occur in proximity to muscles affected by spasticity, botulinum neurotoxin injections can help manage the associated muscle tightness and spasms. By relaxing the overactive muscles, Botox could promote fracture alignment and reduce the risk of displacement during the healing process [30].

In addition, complex fractures often require meticulous reduction techniques for proper alignment. BoNT-induced muscle paralysis could facilitate these reduction manoeuvres, making them more manageable for the surgeon. In this way, by minimising muscle tension, BoNT could aid in achieving accurate and stable fracture reduction.

Further application of BoNT could include prevention of delayed or nonunion, a potential complication in fracture healing [1]. Excessive muscular forces exerted on fracture sites can hinder the formation of a solid union. By temporarily paralyzing the surrounding muscles, Botox could reduce the mechanical stress on the fracture site, optimising the conditions for bone healing and increasing the chances of successful union [6].

When discussing the above potential applications of the neurotoxins, several factors should be considered. One such consideration is the transient nature of its effect [7]. Botulinum neurotoxin-induced muscle paralysis is temporary and reversible, with the effects typically lasting for a few months, depending on the dose and recipient organism before muscle function gradually returns [7, 20, 24]. Therefore, additional interventions, such as physical therapy and rehabilitation, are essential to optimise functional recovery once the paralysis wears off [17].

Moreover, it is important to note that the toxin's applications are not universal [7]. The use of Botox in fracture recovery should be carefully considered, and patient selection should be individualised based on factors such as fracture type, location, associated muscle pathology, and overall patient health [7]. A comprehensive evaluation by a skilled specialist is crucial to determine the appropriate application of botulinum neurotoxins in each case [7, 20].

This experimental study focused on the use of botulinum toxin in the conservative management of clavicular fractures in Wistar rats, tested against a control (NaCl). Points of interest were the physiological response to the injection, X-ray changes in the healing process, and histopathological findings observed in the treated tissues (muscle and bone).

The amount of toxin that was used and the duration of resulting paralysis were determined by a previous experiment, which established through electromyographic testing that 4 IU of botulinum toxin A, administered by intramuscular injection, resulted in paralysis for at least 28 days, without evident toxicity [22]. This second part of the experimental series completes this original research into the effects of BoNT-induced paralysis on clavicle fracture healing.

Radiological evaluation of fracture healing according to the modified Lane–Sandhu scoring system indicated no clear superiority of the toxin in the fracture healing process when compared to controls and no intervention groups. It is noteworthy that in groups A (fracture only) and D (fracture plus saline), there was one incidence of nonhealing, respectively. These isolated findings are not sufficient to prove any causal relationship between the toxin and the degree of healing achieved but are worth exploring further. Overall, all three fracture groups showed variable results, mostly of average healing, with one animal in each group achieving complete healing.

The histological scoring system by Huo et al. was used for conducting a histopathological evaluation of the healing process [28]. This showed no significant differences between the experimental and control groups, suggesting that the toxin's administration on the sternocleidomastoid muscle did not affect the healing process of clavicle fractures.

Of interest were other findings of the histological evaluation of the muscle tissue adjacent to the fractured bone between the experimental and control groups. Lesions observed in STM muscles injected with the toxin were more pronounced than in the other groups. Overall, tissue injected with botulin showed more enhanced cell growth and hypertrophy, also necrosis and atrophy, as opposed to the more subtle changes observed in untreated tissue or tissue injected with saline.

These findings are important to acknowledge as potential limitations or even adverse consequences secondary to botulinum neurotoxin A application. Literature supports the idea that such effects can be long-term and potentially not reversible, even after the toxin's paralytic effect subsides [31]. A study on healthy volunteers reviewing muscle treated with Botox against saline controls through magnetic resonance imaging, histochemistry, and electron microscopy indicated that muscle changes were sustained over twelve months and included neurogenic atrophy and degenerative changes of the neuromuscular junction [32]. A different study, conducted on animal subjects, indicated that repeated administration of the toxin on muscle groups resulted in decreased muscle torque compared to muscles treated only once [31].

Postinjection manifestations reviewed in therapeutic applications of the BoNT in children treated for cerebral palsy showed muscle volume loss, sustained for at least six months [33]. At the same time, other research has shown that not only does paralysis not aid fracture healing, but it also interferes with it by causing apoptosis and bone degradation [8, 34, 35].

The present study is the first to the authors' knowledge to investigate the effects of botulinum neurotoxin A on the conservative healing of clavicle fractures in animals and one of the few experimental research projects in animal subjects to investigate the effects of the toxin on muscle tissue. A variety of methods were used for the visualization and review of the tissue, including neurophysiologic, radiographic, and histopathologic control, offering a plethora of results, and aiding in a better understanding of the toxin's effects. Although the results of the study do not support the use of the toxin in the conservative treatment of fractures, they offer valuable insights into the prevention of the toxin's use to avoid unnecessary and potentially risky interventions with increased costs.

Although the number of animals used was small, this was a decision made on the balance of ethics aiming to keep their numbers to a minimum, while also producing meaningful results. In addition, the inability to achieve compliance with immobilization in animals is an important limitation to consider when reviewing the results of callus formation and evidence of histopathological healing of the fracture. Imaging was not obtained during the healing period but was instead only completed at the end of the treatment period on day 28, as complete healing is expected to have occurred on healthy rats by this date [37]. Although more frequent imaging of the gradual healing process could have provided additional data, this decision was based on the risk versus benefit of putting the animals through general anaesthesia, something which could in turn result in deaths. Finally, fracture union and healing would have been better visualized by additional imaging, such as computed tomography [38]. This can be considered in future research.

While the applications of botulinum neurotoxins continue to primarily concern aesthetics, therapeutic functions of the toxin are expanding. With this in mind, more research is required to investigate the risks and benefits of the toxin's application in this context, as well as the long-term health sequelae.

## Figures and Tables

**Table 1:** Group allocation of animals according to intervention.

Groups	Animals in group	Intervention
Group A	7	Clavicle fracture only
Group B	7	4 IU toxin only
Group C	7	Clavicle fracture + 4 IU toxin
Group D	7	Clavicle fracture + 0.9% NaCl

## Data Availability

The radiological and histology data used to support the findings of this study are included in the article.
